# Selective eradication of cancer cells by delivery of adenovirus-based toxins

**DOI:** 10.18632/oncotarget.16934

**Published:** 2017-04-07

**Authors:** Shiran Shapira, Assaf Shapira, Diana Kazanov, Gil Hevroni, Sarah Kraus, Nadir Arber

**Affiliations:** ^1^ Laboratory of Molecular Biology, The Integrated Cancer Prevention Center, Tel Aviv Sourasky Medical Center, Affiliated to the Sackler Faculty of Medicine, Tel Aviv University, Tel-Aviv, Israel; ^2^ Department of Molecular Microbiology and Biotechnology, The George S. Wise Faculty of Life Sciences, Tel Aviv University, Tel-Aviv, Israel

**Keywords:** MazEF, toxin-antitoxin, KRAS mutation, cancer gene therapy

## Abstract

**Background and objective:**

*KRAS* mutation is an early event in colorectal cancer carcinogenesis. We previously reported that a recombinant adenovirus, carrying a pro-apoptotic gene (*PUMA*) under the regulation of Ets/AP1 (RAS-responsive elements) suppressed the growth of cancer cells harboring hyperactive KRAS. We propose to exploit the hyperactive RAS pathway, rather than to inhibit it as was previously tried and failed repeatedly. We aim to improve efficacy by substituting *PUMA* with a more potent toxin, the bacterial MazF-MazE toxin-antitoxin system, under a very tight regulation.

**Results:**

A massive cell death, in a dose-dependent manner, reaching 73% at MOI 10 was seen in KRAS cells as compared to 22% in WT cells. Increase expression of MazE (the anti-toxin) protected normal cells from any possible internal or external leakage of the system and confirmed the selectivity, specificity and safety of the targeting system. Considerable tumor shrinkage (61%) was demonstrated *in vivo* following MazEF-encoding adenovirus treatment without any side effects.

**Design:**

Efficient vectors for cancer-directed gene delivery were constructed; “pAdEasy-Py4-SV40mP-mCherry-MazF”“pAdEasy-Py4-SV40mP-mCherry-MazF-IRES-TetR-CMVmp-MazE-IRES-EGFP“,“pAdEasy-ΔPy4-SV40mP-mCherry-MazF-IRES-TetR-CMVmp-MazE-IRES-EGFP “and “pAdEasy-mCherry”. Virus particles were produced and their potency was tested. Cell death was measured qualitatively by using the fluorescent microscopy and colony formation assay, and was quantified by MTT. FACS analysis using annexin V and RedDot2 dyes was performed for measuring apoptotic and dead cells, respectively. *In vivo* tumor formation was measured in a xenograft model.

**Conclusions:**

A proof of concept for a novel cancer safe and effective gene therapy exploiting an aberrant hyperactive pathway is achievable.

## INTRODUCTION

Colorectal cancer (CRC) is a major health concern in the Western world [1]. The prognosis for metastatic CRC still remains unsatisfactory. Resistance to chemotherapy is a major obstacle for effective treatment. CRC patients carrying *KRAS* mutations are of particular therapeutic challenge, due to their resistance to anti- EGFR therapies.

Aberrant activation of the RAS pathway plays an important role in the multistep process of CRC carcinogenesis. Oncogenic RAS stimulates a number of downstream effectors that activate several transcription factors that bind to the RAS-responsive DNA element and induce early response gene expression. The polyoma (Py) virus enhancer consists flanking overlapping binding sites of the Ets and AP1 transcription factors that are essential for oncogene transcriptional activation [2].

Viral gene therapy is an innovative approach that offers a potential treatment for inherited and acquired diseases It usually involves generating the replication of defective viral particles that are capable of stably or transiently introducing a desirable transgene into cells. This ultimately results in the slow progression of cancer cell growth [3–5]. The most characterized human adenoviruses of serotypes 2 and 5 (Ad2 and Ad5, respectively) usually cause mild upper respiratory tract infections, making them well suited for use in gene therapy.

Adenovirus-based cancer therapy is used for two main strategies: (i) direct tumor cell killing through the delivery of replicating oncolytic viruses, or via non-replicating vectors encoding tumor suppressor genes, suicide genes or anti-angiogenic genes, (ii) destroy primary and metastatic cancer cells through induction of host antitumor immune responses [6]. These approaches offer potential for selective tumor cell destruction without damage to normal tissues. Apoptotic tumor suppressor genes are used extensively in this field [7], either alone or in combination with chemotherapy. However, the ability to specifically target tumor cells with gene transfer is limited, and many normal cells are often affected as well.

Previous studies in our laboratory have shown that recombinant adenovirus carrying the lethal gene *PUMA* (p53-upregulated modulator of apoptosis) (generous gift of Bert Vogelstein, Johns Hopkins University, Baltimore) under the control of Ets and AP1-RAS-responsive elements (Py2-SV40-PUMA) suppressed the growth of a variety of tumor cells harboring mutated RAS [8–13]. We have also recently shown that the addition of multiple RAS-responsive elements (Py4/Py5-SV40-PUMA) further improved the growth inhibitory potency of the construct and induced apoptosis in CRC and pancreatic cancer cells *in vitro* and *in vivo* [12, 14]. However, escape mechanisms are likely to arise, and the cancer cells will increase the expression of anti-apoptotic genes, rendering the cells resistant as the induced programmed cell death pathway will be inactivated. Herein, we suggest that tightening the expression of the toxin and replacing the pro-apoptotic gene by a significantly more potent toxic molecule that does not exist in human cells will serve as an improved approach. MazF is a bacterial ribonuclease known to have specificity for ACA sequences in single-stranded RNA. MazF-induced toxicity is executed by blocking *de novo* protein synthesis through its endoribonuclease activity, termed mRNA interferases [15]. In nature, MazF is one of a pair of genes encoding for a stable toxin and an unstable antitoxin organized in a bicistronic operon as a part of a flexible genome [16]. The antitoxin interferes with the lethal action of the toxin and neutralizes its toxicity [17, 18]. This organization is a hallmark of toxin–antitoxin (TA) operons. TA systems are evolutionarily successful entities that are prevalent in lower organisms such as bacteria and archaea, and they play important roles in a diverse range of cellular activities [19]. While some TA systems are found exclusively in plasmids, others integrate into host regulatory networks (encoded from the chromosome). The first identified TA system was shown to play a role in plasmid maintenance [20]. Once a cell loses the plasmid encoding the TA system, the toxin is released from the existing TA complex, given that the antitoxin is more unstable than the toxin. This results in cell growth inhibition that eventually leads to cell death [21].

Herein, an innovative and more regulated TA system derived from *E. coli* enables selective control and efficient killing of tumor cells while sparing normal cells.

## RESULTS

### Eradication of mutated *RAS*–harboring cells by adenovirus-mediated delivery of MazF ribonuclease

**Figure 1:**
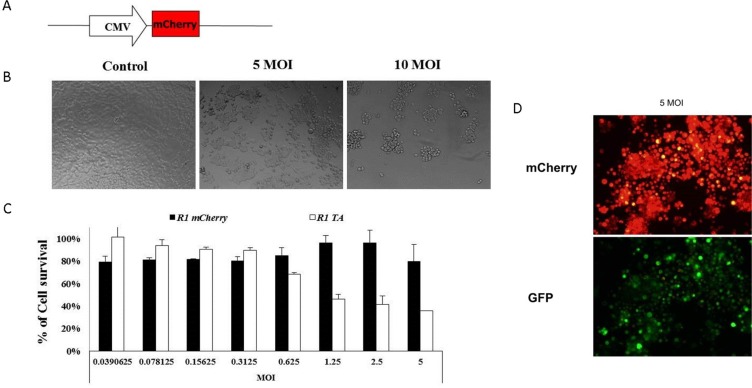
Schematic illustration of the mazF cassette (**A**) The RRE-activated MazF cassette was constructed by cloning several elements in the following order (from the N terminus): four repeats of the RAS responsive, Ets and AP-1 binding sites, Py2; SV40 minimal promoter; monomeric red fluorescence protein mCherry; *E. coli* MazF ribonuclease. (**B**) The toxic effect of the mazF-encoding viruses. 1 × 10^4^ HCT116 cells were seeded in 96-well plates in complete medium. Median dilutions of the MazF-encoding viruses starting from 25 MOI were added to the cells on the next day. (B, **C**) Cell survival was evaluated by microscope examination (10 μm) (**D**) and by enzymatic MTT assay72 hours after infection. Each bar represents the mean ± SD of a set of data determined in triplicates. (**E**, **F**). 1 × 10^5^ cells were seeded in 12-well plates in complete medium and infected with the different adenoviruses in 20 MOI for 72 hours. Cell death was measured by FACS after staining with Annexin V (E) and RedDot2 (F) dyes.

The potency and ability of MazF to kill the target cells were evaluated prior to engineering a more complex system with several toxicity control points. Massive cell death, in a dose-dependent manner, was induced following infection of HCT116 cells [containing a mutated *KRAS* at codon 13 (Gly to Asp)] with Ad-Py4-SV40-mCherry-MazF (Figure 1A). Figure 1 shows the cytotoxicity induced by the ribonuclease activity that was qualitatively evaluated by a fluorescent microscope examination 72 hours after the infection (Figure 1C) as compared to the uninfected cells (Figure 1B). About 35% cell survival (relative to the uninfected controls) was quantitatively measured by the enzymatic MTT assay upon treatment, when employing a MOI of 25 (Figure 1D). Cytotoxic activity of MazF was confirmed by FACS analysis: 50% apoptosis was measured using annexin V (Figure 1E), while about 80% membrane compromised or dead cells was detected with RedDot2 (Figure 1F).

### Rational design of an innovative toxin-antitoxin cassette for enhanced regulation

**Figure 2:**
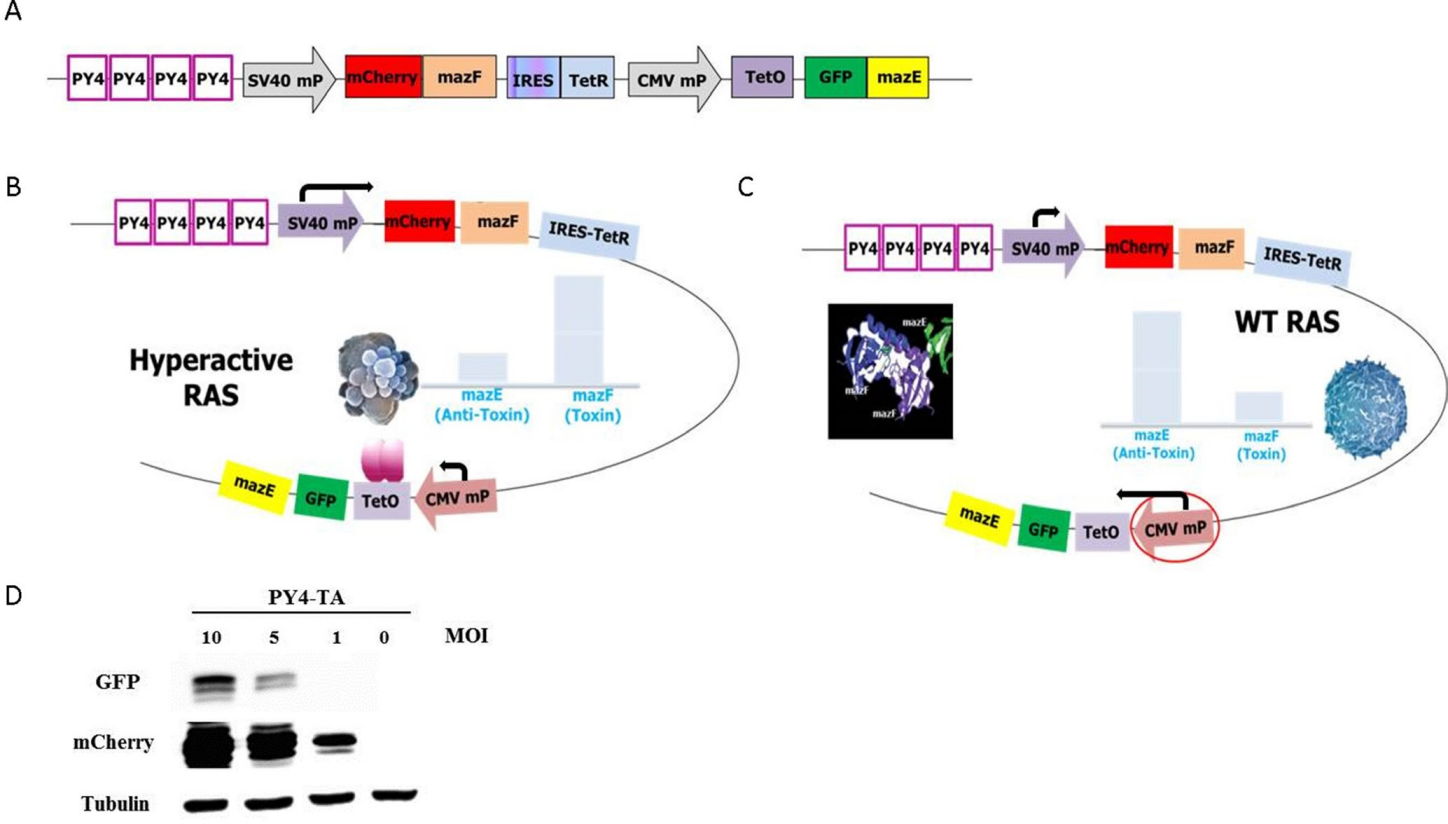
Schematic illustration of the mazF cassette (**A**) The RRE-activated MazEF cassette was constructed by cloning several elements in the following order (from the N terminus): four repeats of the Ras responsive Ets and AP-1 binding sites, Py2; SV40 minimal promoter; monomeric red fluorescence protein mCherry; *E. coli* MazF ribonuclease; internal ribosome entry sites; tetracycline repressor coding sequence; CMV minimal promoter; tetracycline operator; green fluorescence protein; *E. coli* antitoxin MazE. (**B**) The PY4 element increased the presence and activity of the MazE in the cells harboring hyperactive RAS and in the cells doomed to die. (**C**) The activity of MazF was inhibited in cells that displayed WT *RAS*, and the cells were protected and saved from its toxicity, thus ensuring their survival. (**D**) A representative western blot that confirms the differences in the degree of expression of the toxin (represented by the mCherry protein) vs. antitoxin (represented by the GFP protein).

The rationale behind the design of the“pAdEasy-Py4-SV40Mp-mCherry-MazF-IRES-TetR-CMVmp-MazE-IRES-EGFP“construct (Figure 2A), or briefly, “pAdEasy-Py4-TA”, was to couple the ribonuclease activity with its antidote in order to enable protection of non-target cells (i.e., normal cells without a hyperactive RAS pathway) while allowing a high level of expression of the toxic agent in mutated *RAS*-harboring cells.

In hyperactive RAS cells (Figure 2B), the Py4 enhancer element induced toxin expression significantly more than that of the antitoxin. Figure 2D shows a representative western blot that confirms the differences in the degree of expression of the toxin vs. antitoxin. The Tet repressor, which is also expressed in high levels, binds to the Tet operator sequence and further inhibits the transcription of the antitoxin. Altogether, MazF is expected to overcome the antitoxin inhibition and the cells are expected to die.

In cells that do not harbor mutated *RAS* (Figure 2C), the Py4 enhancer is not activated, and therefore there is no preference for expression from the SV40 mP. Since the CMV mP is slightly stronger than the SV40 mP and one molecule of AT inhibits two molecules of toxin, the inhibitory activity of the antitoxin should prevail. Consequently, the MazE in these cells will overcome the toxicity of MazF and the cells will survive.

### MazE protects normal cells from MazF cytotoxic activity

**Figure 3:**
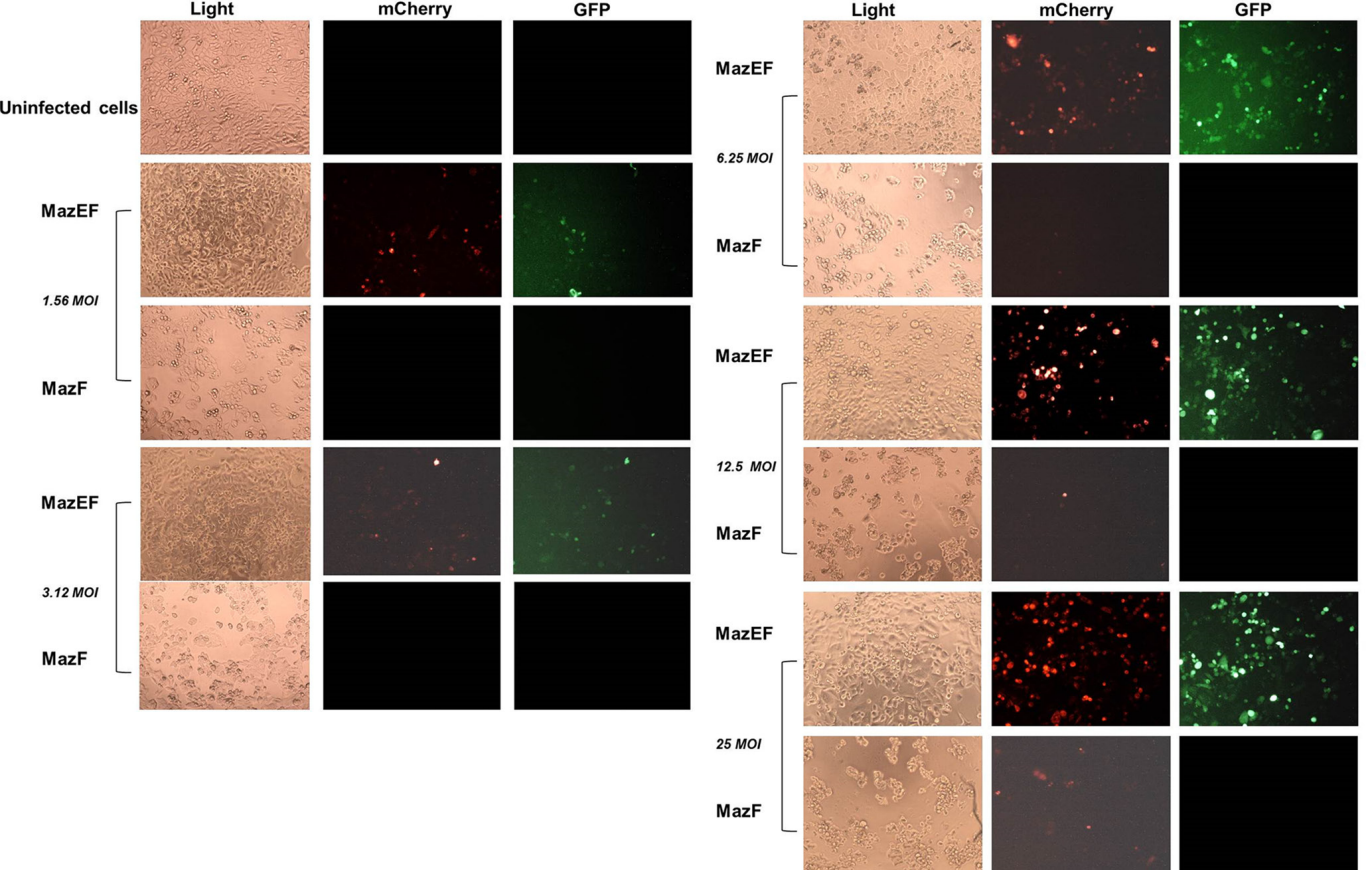
Normal cells are protected from the cytotoxicity of MazF due to MazE expression HT29 cells were seeded in 96-well plates. After 24 hours, two-fold dilutions of recombinant adenoviruses encoding for MazF or MazEF were added for 72 hours. Qualitative examination was performed using light and fluorescence microscopy (10 μm).

In order to demonstrate the advantage of using the pAdEasy-Py4-TA cassette, we tested its ability to protect cells with wild type (WT) *RAS* from possible “leakage” of the lethal gene. The basal expression from the SV40 mP along with low expression levels of RAS in normal cells induces low expression of MazF. However, even this low level of expression is sufficient to kill a cell. HT29 cells, with WT *RAS*, were infected with twofold dilutions of the MazEF- or MazF-encoding viruses, and the viability of the cells was qualitatively examined by light and fluorescence microscopy. As shown in Figure 3, infection with MazF decreased cell viability, indicating a leakiness of MazF expression even in the absence of mutated *RAS*. In contrast, infection with the MazEF construct was well tolerated. When visualized under a fluorescence microscope, the intoxicated Ad-Py4-SV40-mCherry-MazF-infected cells showed very faint red fluorescence, indicating inefficient mCherry-MazF accumulation. This is due to the ribonuclease activity of MazF that results in inhibition of protein synthesis, including its own [22, 23]. On the other hand, the ribonuclease activity of MazF was neutralized by its antidote MazE in cells infected with pAdEasy-Py4-TA, as indicated by the presence of both red and green fluorescence (Figure 3).

### Adenovirus-mediated delivery of TA-encoding cassette specifically eliminates R1 cells harboring activated RAS

**Figure 4:**
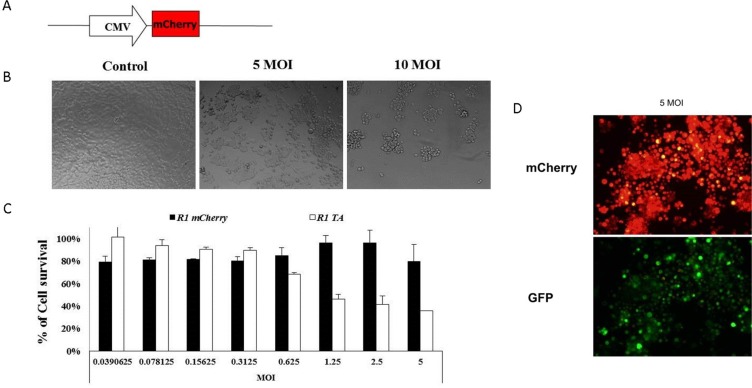
Eradication of R1 cells by recombinant adenovirus-mediated delivery of the MazF-MazE encoding cassette (**A**) The mCherry control cassette was constructed by cloning the monomeric red fluorescence protein mCherry downstream to the CMV minimal promoter. 1 × 10^4^ R1 cells were seeded in 96-well plates. After 24 hours, two-fold dilutions of recombinant adenoviruses encoding for MazEF or mCherry were added for 72 hours. (**B**) Light and (**D**) fluorescence microscopic examination (10 μm) (**C**) and enzymatic MTT viability assay were performed 72 hours post-infection. (B) Representative pictures of uninfected cells and cells that were infected (with the MazEF cassette) with 5 and 10 MOI, (D) and representative fluorescence pictures of the infected cell (5 MOI of MazF encoding viruses). The relative fraction of viable cells (relative to uninfected controls) was determined by MTT assay. (C) Each bar represents the mean ± SD of a set of data determined in triplicates.

The potential of the MazEF-encoding cassette to kill target cells was tested in a proof of concept study performed in R1 cells, which serve as a model system for hyperactive RAS-harboring cells [24]. R1 cells were infected with twofold dilutions of the mCherry (Figure 4A)- or MazEF-encoding viruses. Infection of R1 cells with MazEF elicited a considerable cytotoxic effect, decreasing viability to 36% (at MOI 5) relative to the uninfected controls, while no significant effect was seen after infection with the mCherry cassette (80–90%) (shown qualitatively by light microscope in Figure 4B and quantitatively by MTT in Figure 4C). The expression of the GFP and mCherry proteins indicates that both, MazF and MazE, components had been expressed (Figure 4D). However, as we hypothesized and demonstrated in Figure 4B–4D, the MazF cytotoxic activity was not inhibited; the labile antitoxin failed to accumulate, and the Py4 element induced higher expression of MazF compared to that of MazE.

### The MazEF cassette kills mutated RAS-harboring cells

**Figure 5:**
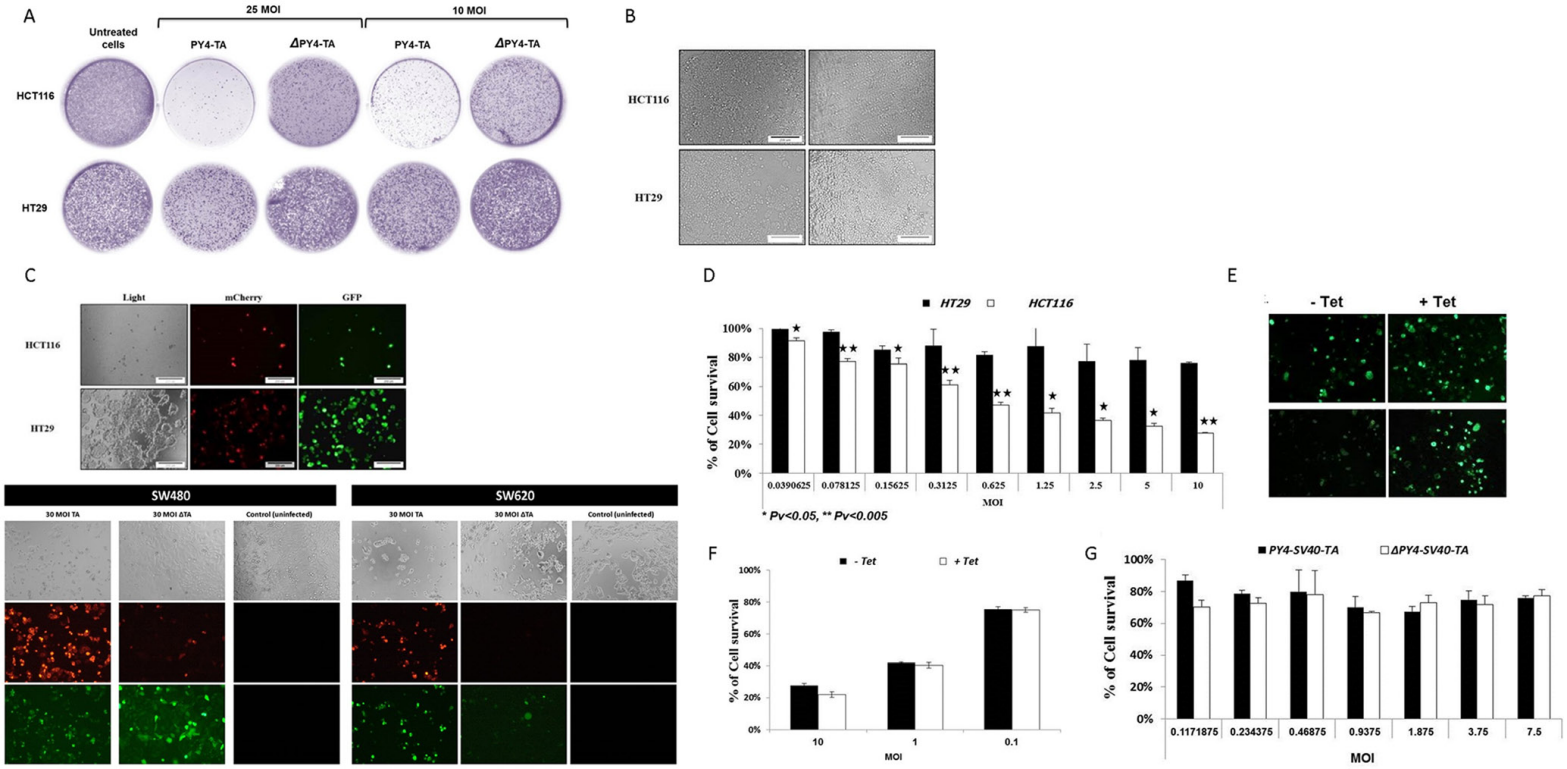
Selective eradication of CRC cells by recombinant adenovirus-mediated delivery of the mazEF encoding cassette (**A**) Colony formation assay. On the day before infection, 5 × 10^5^ HCT116 and HT29 cells were seeded in 6-well plates and subsequently infected with 25 and 10 MOI of the viruses “pAdEasy-Py4-mCherry-MazF-IRES-TetR-CMVmp-MazE-IRES-EGFP“ and “pAdEasy-SV40-mCherry-MazF-IRES-TetR-CMVmp-MazE-IRES-EGFP” or left un-infected. After 7 hours, the cells were trypsinized and seeded at 3-fold dilutions and incubated for 7 days. Surviving colonies were stained with 0.02% crystal violet. (**B**–**F**) 1 × 10^4^ HCT116, SW480, SW620 and HT29 cells were seeded in 96-well plates. After 24 hours, two-fold dilutions of recombinant adenoviruses encoding for mazEF were added. Microscopic examination of the (B) uninfected and (C) infected cells (10 MOI) was performed 72 hours post-infection. (D) The results (HT29 and HCT116 as representative cell lines) were verified by the enzymatic MTT viability assay, and the relative fraction of viable cells (relative to uninfected controls) was determined. Each bar represents the mean ±SD of a set of data determined in triplicates. (E) HT29 cells were infected (10 MOI) in the presence and absence of 1 μg/ml tetracycline. The cells were examined microscopically 72 hour post-infection. (F) 1 × 10^4^ HCT116 cells were seeded in 96-well plates. The Ad-Py4-TA encoding viruses were added in several MOIs with or without 1 μg/ml tetracycline 24 hours later. The enzymatic MTT viability assay was performed after 72 hours. (**G**) 1 × 10^4^ HT29 cells were seeded in 96-well plates. After 24 hours, two-fold dilutions of recombinant adenoviruses (PY4-SV40-mazEF and ΔPY4-SV40-mazEF) were added for 72 hours. Then, the enzymatic MTT viability assay was performed, and the relative fraction of viable cells (relative to uninfected controls) was determined. Each bar represents the mean ± SD of a set of data determined in triplicates.

An *in vitro* colony-forming assay was performed to qualitatively and comparatively assess the sensitivity of CRC cells with mutated *RAS* to the expression of the transgene. In addition, this assay was intended to verify that MazF is well tolerated by the normal cells that do not harbor hyperactive RAS. HCT116 (harboring hyperactive RAS) and HT29 (no *KRAS* mutation) cells were infected with 25 and 10 MOI of “pAdEasy-Py4-TA“, “pAdEasy-ΔPy4-TA” or left uninfected. The cells were trypsinized and seeded in 3-fold dilutions 7 hours later. Surviving colonies were stained after 7 days. Figure 5A shows the potency of the expressed transgene under the control of the RRE compared to uninfected cells and to the control cassette carrying a deletion of the RRE. Prominent differences in the numbers of surviving colonies were observed, confirming that MazF indeed overcame the inhibitory effect of MazE in mutated RAS-harboring cells while MazE was able to protect normal cells from MazF toxicity. In addition, the selectivity of our targeting system was confirmed since the massive cell death took place only in the RRE- including cassette, while no significant effect was seen after infection with the ΔPy4-TA cassette that lacks the RRE.

### Adenovirus-mediated delivery of TA encoding cassette specifically eliminates activated RAS-harboring CRC cells

We further examined the ability of the above-described TA system to kill a human cancer cell line expressing hyperactive RAS. HCT116 cells were infected with twofold dilutions of the MazEF-encoding viruses, starting from 10 MOI (Figure 5C), or left untreated (Figure 5B). Massive cell death (73%, relative to the uninfected control, at 10 MOI) was demonstrated exclusively in the mutated *KRAS* harboring cells, emphasizing the potency of this system (Figure 5D). The percentage and intensity of the fluorescence of green cells was higher than that of the red ones in HT29 cells, lacking hyperactive RAS (Figure 5C). This indicates that the expression of the antitoxin increases and exceeds that of the toxin in normal cells. In order to confirm and further support the ability of this system to protect normal cells on one hand, and to efficiently kill cancer cells on the other hand, additional CRC cell lines (such as SW620, SW480 and DLD1) were tested and yielded very similar results, substantiating the above observations (Figure 5C). The potency of this system was also tested for other cancer types, such as pancreatic and prostate, suggesting a wide range of therapeutic potential of this suggested treatment modality (data not shown). Moreover, inclusion of tetracycline provided an additional protective layer, not only for virus production and propagation but also for normal cell protection. Binding of tetracycline to the Tet repressor led to a conformation change that resulted in relief of the MazE transcriptional inhibition. Consequently, expression of the antitoxin was increased in naïve cells, as demonstrated by the increase in green fluorescence (Figure 5E), while tetracycline did not compromise the toxicity of MazF in mutated *RAS-*harboring cells (Figure 5F).

In addition, cell viability assay (Figure 5G) that quantifies the effect of PY4-TA on HT29 cells compared to the control, ΔPY4-TA, shows that no significant effect on cell survival was observed after infection with the ΔPY4-TA cassette.

### Deletion of the RAS-responsive element decreases the cytotoxic activity of MazF

**Figure 6:**
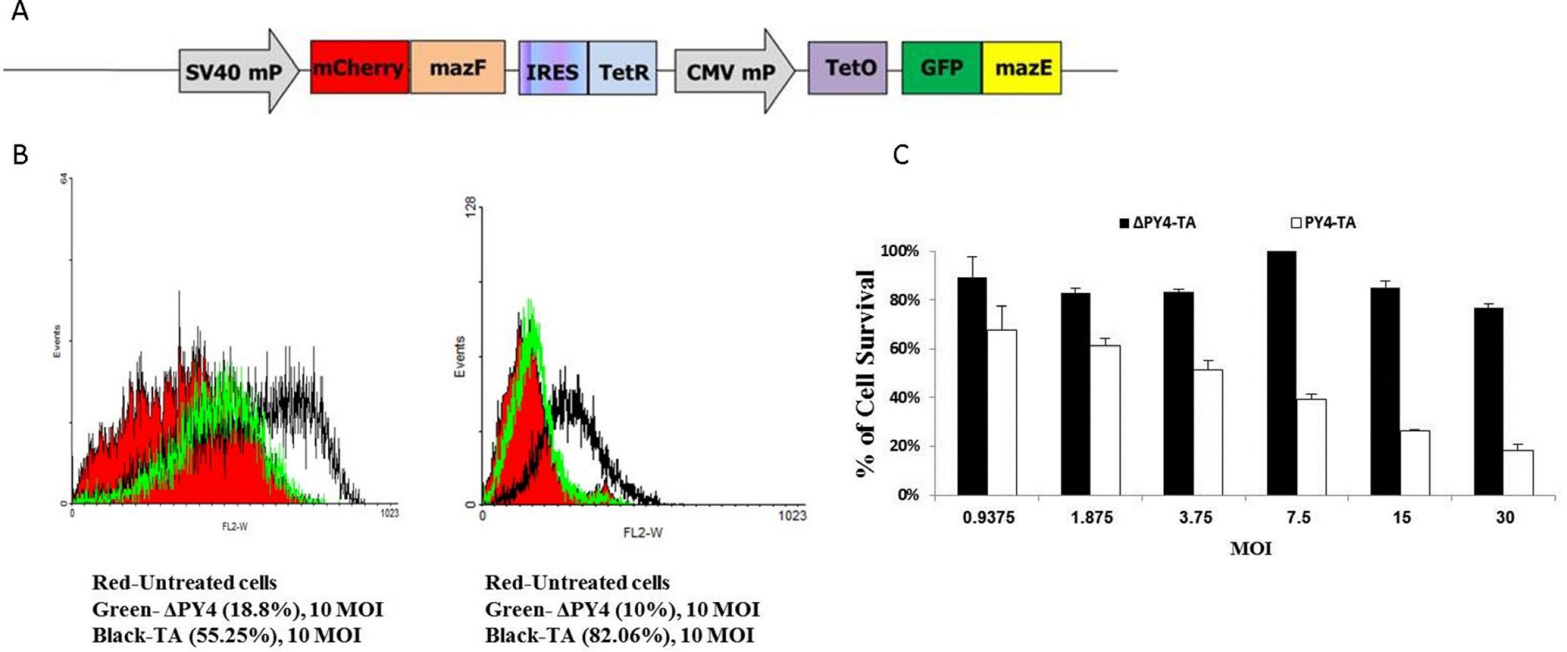
Deletion of the RAS responsive DNA element (Py4) (**A**) Schematic illustration of the control cassette that lacks the RRE. (**B**) 1 × 10^5^ HCT116 cells were seeded in 12-well plates in complete medium and infected with the different adenoviruses at 10 MOI for 72 hours. Cell death was measured by FACS after staining with Annexin V and RedDot2 dyes. (**C**) The untreated cells are shown in red, the toxin antitoxin-treated cells are shown in black, and the cells that were treated with the RRE deletion cassette are shown in green. 1 × 10^4^ cells were seeded in 96-well plates. After 24 hours, two-fold dilutions of recombinant adenoviruses encoding for Py4-TA or ΔPy4-TA were added for 72 hours. The relative fraction of viable cells (relative to uninfected controls) was determined by MTT assay. (C) Each bar represents the mean ± SD of a set of data determined in triplicates.

As mentioned above, an additional cassette lacking the RRE, “pAdEasy-ΔPy4-TA”, was also constructed (Figure 6A). Figure 6 demonstrates that deletion of the RAS-responsive DNA element enhanced the contribution of MazE and therefore improved the selectivity of this targeting system. Cell viability was measured by FACS analysis: while massive cell death (55% apoptosis, 82% dead cells) was observed following infection with the full toxin-antitoxin encoding viruses, deletion of the RAS-responsive DNA element dramatically reduced this effect to 18% and 10%, respectively (Figure 6B). These results were also confirmed by the enzymatic MTT assay (Figure 6C), where a difference of about 60% was observed between Py4-TA and ΔPy4-TA.

### MazEF-encoding viruses inhibit tumor growth *in vivo*

**Figure 7:**
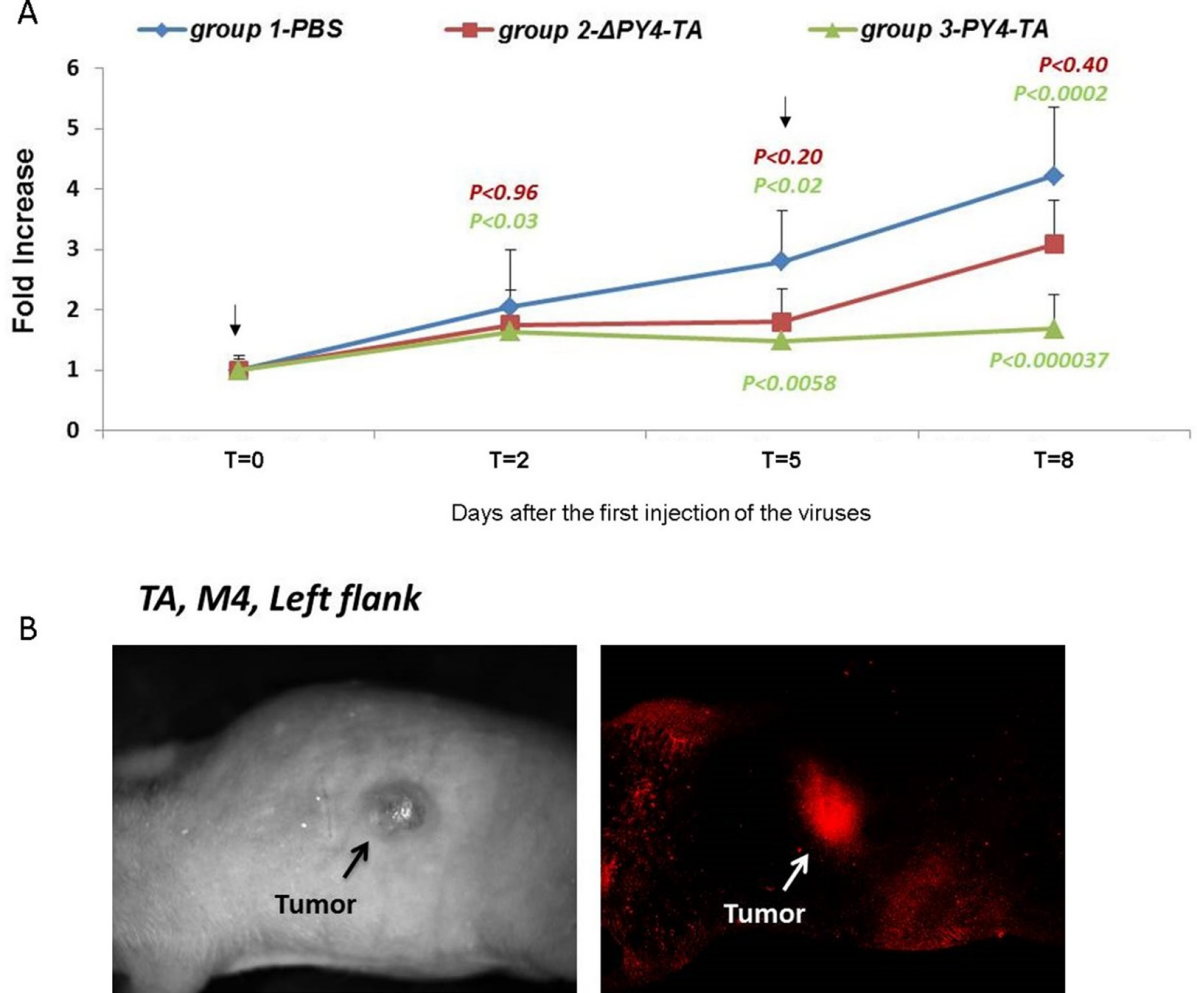
Inhibition of tumor growth in mice Tumors were formed in nude mice by subcutaneous injection of 5 × 10^6^ HCT116 cells on day 0 and were treated twice with intraperitoneal 2 × 10^9^ PFU/mouse. (**A**) Tumor size was measured at the indicated time points and tumor volumes were calculated. The mean values for each group are shown, and the standard deviation is represented by error bars for each measurement. The *P* values for the ΔPy4-TA group compared to the PBS group are shown in red and those for the Py4-TA group compared to the PBS group are shown in green. Each bar represents the mean ± SD of a set of data determined from six mice. (**B**) Imaging was performed on the living organism with the Maestro CRi imaging device and outside the mouse body. The red fluorescence dye represents the expression of MazF and the green fluorescence dye represents the expression of MazE.

The therapeutic potential of the TA system was tested by specific targeting of tumor cells in nude mice bearing a xenograft of HCT116 CRC cells, harboring mutated *RAS*. The growth of these cells was markedly inhibited by Py4-TA-encoding viruses (Figure 7A). Impressive tumor shrinkage was demonstrated *in vivo* following treatment with Ad-Py4-SV40-MazEF-encoding adenovirus (61%) (*P <* 0.0002) without producing any toxic side effects. In the Ad-ΔPy4-SV40-MazEF treated mice (control group) tumor volume was reduced by only 27% (*P <* 0.4). No growth inhibition was seen following injection of PBS (Figure 7A).

Throughout this study the mice were monitored for their general well-being, weight, and food and water consumption. At no time was there any indication of toxicity ascribable to the adenoviral vectors (data not shown).

The expression of MazF and MazE in the infected tumor cells was monitored with the Maestro imaging CRi device (Figure 7B). The imaging was performed on live mice (Figure 7B) and outside the mice body (data not shown). This analysis confirmed that the adenoviruses indeed targeted the mutated *RAS*-harboring tumors and that the MazEF genes were expressed.

## DISCUSSION

Selective eradication of cancer cells using adenovirus as a vehicle for toxins and anti-toxins delivery is shown herein to be effective and safe. It exploits, rather than trying to inhibit, the active pathway that transformed the normal cell into a malignant one. This approach may overcome the major obstacle of chemotherapy, which is toxicity to normal organs and tissues.

The use of a hyperactive RAS pathway is a promising novel therapeutic option in cancer therapy. The concept of exploiting the *RAS* mutation with pro-apoptotic genes, under the control of the RRE-mP, was already shown to be successful by our group [8, 12, 14]. Herein, the constructs were scaled up for a tighter control of the toxin level and to overcome any potential leakage of the system. With tight control of the toxin and anti-toxin expression, it was possible to replace the mild lethal gene, *PUMA*, with significantly more toxic agents. Enzymes that inhibit protein synthesis, such as the bacterial MazF protein, represent some of the most potent agents for killing cells.

When using more potent toxins, the leakiness from the minimal promoter alone can be sufficient to cause significant cell death. The innovative system provides additional internal regulations that can prevent this problematic issue. Herein, a natural system that regulates cell death in a lower organism, *E. coli*, was used.

Normally, TA systems consist of toxic proteins that disrupt cellular processes along with labile antitoxins that counteract this growth inhibition. One of the most thoroughly studied toxin–antitoxin systems, the *E. coli* MazEF chromosomal module was selected. Massive cell death in mutated *RAS* cells was achieved following MazF expression, while its leakage into wild-type *RAS* cells was controlled by the antitoxin expression. Several layers of regulations were added to this unique cassette, including the MazE anti-toxin, the tetracycline repressor, the tetracycline operator, and the CMV mP. They have a great impact on the balance of the level of expression of these two components in the absence of the Py4 element or when the virus infects normal cells (carrying WT *KRAS*). Furthermore, for cell death, confirmation of the annexin V staining was done by PARP-1 cleavage (data not shown). PARP-1, involved in DNA repair, is a well-known substrate for caspase-3 cleavage during apoptosis. Its cleavage is considered to be a hallmark of apoptosis and we have shown that the expression of MazF in *KRAS* mutated cells resulted in its cleavage.

Toxin-antitoxin systems are currently being examined for possible applications. MazF is used in the therapy of HIV and HCV [23, 25, 26]. The potential use of MazF in cancer was recently reported [27]. However, since both normal and tumor cells are sensitive to MazF, it is essential to develop specific and effective expression and delivery systems for MazF exclusively in tumor cells.

Noteworthy, the replacement of the pro-apoptotic agent with a more lethal one complicates the production of the viruses since the leakage of toxin expression in the packaging cells leads to lower virus titers. A new packaging cell line that constitutively expresses the anti-dote was established (S2). However, the use of the toxin-antitoxin system allows the production of viruses in the common packaging cells. During virus production, tetracycline was added to the growth media in order to overcome the inhibition of MazF on transcription.

The adenoviruses are among the most commonly used vectors for the delivery of genetic material into human cells. The main use of adenoviral vectors is in suicide gene therapy, gene-based immunotherapy, gene replacement strategies, and in combination with chemotherapy. Adenoviruses have limitations, the most important one being their immunogenicity that precludes more than one effective injection. Therefore, we are currently developing adeno-associated virus (AAV)-based vectors that lack immunogenicity and carry the TA cassette.

Herein, we showed that expression of our cassettes (encoding for MazF and MazEF) resulted in massive tumor cell death. Interestingly, the presence of MazE in the MazEF cassette did not decrease the potency of the treatment. Contrary, infection with the MazEF viruses resulted in a greater killing effect compared to that of the MazF viruses. A possible explanation for this is that the transcript of the MazF from the Py4-SV40-mCherry-MazF cassette is different from the Py4-TA cassette and therefore affects the stability of the mRNA and leads to different expression levels. In addition, the presence and proximity of the CMV mP, and consequently, the transcription factors that are recruited to it, can reinforce the SV40 mP activity and lead to higher MazF expression levels. Most cancer gene therapy protocols involve combinations with chemotherapy. The high efficacy of the current method may be used as a single agent.

The antitumor effect of the Py4-SV40-TA *in vivo* is impressive, even after being administrated only twice (Figure 7). The lack of toxicity holds promise for effective therapy in devastating human malignancies with an active RAS pathway (pancreatic, lung, melanoma and colon cancer). We are currently evaluating the potency of our system in a more clinical model for colorectal cancer, an orthotropic model, where the cells will be inoculated to the colon of the mice and tumor volume will be measured by colonoscopy.

## MATERIALS AND METHODS

All reagents were purchased from Sigma, Israel unless otherwise stated. All secondary HRP-conjugated antibodies were purchased from Jackson ImmunoResearch Laboratories, USA. ECL reagent, cell culture media, and additives were from Beit-Haemek, Israel. Nitrocellulose filters were purchased from Schleicher & Schuell BioScience, USA. Annexin V and Reddot2 dye were purchased from Biotium, and G418 was purchased from Gibco. All plasmid and DNA fragment purifications were carried out with a High-Speed Plasmid Mini Kit and a Zymoclean^TM^ Gel/PCR DNA recovery Kit (Fermentas and Zemo Research, respectively) unless otherwise specified. T4 DNA ligase and restriction enzymes were purchased from New England Biolabs, USA. DNA ligations were carried out overnight at 16^°^C.

### Bacterial strains

The following *Escherichia coli* (*E. coli*) strains were used: DH5a (Stratagene, USA) for plasmid propagation and BJ5183 (Stratagene, USA) for the generation of recombinant adenovirus plasmid DNA.

### Cell lines

HT29 human colon adenocarcinoma, HCT116 human colon cancer, R1 KRAS transformed rat enterocytes and HEK293 human kidney cell lines were grown in high-glucose Dulbecco's modified Eagle's medium (DMEM), all supplemented with 5% heat-inactivated fetal bovine serum (FBS), 1% penicillin and streptomycin in an atmosphere of 95% oxygen and 5% CO_2_. 1 μg/ml tetracycline was added to the HEK293 medium for TA virus production. In addition, 600 μg/ml G418 was added to the culture medium of MazE-expressing cells.

### Construction and propagation of recombinant adenovirus vectors

*Oligonucleotides*. All the oligonucleotides that were used in this study were purchased from Sigma, Israel. Recombinant DNA techniques were carried out according to standard protocols or as recommended by the suppliers. A more detailed description of the procedure is provided in Supplementary Text 1.

### Cell-viability assay

The cell-killing activities of adenoviruses encoding for MazF, and MazEF were measured by the Thiazolyl Blue Tetrazoliam Bromide (MTT) enzymatic assay. Briefly, 1 × 10^4^ cells were seeded in 96-well plates. After 24 hours, different dilutions of recombinant adenoviruses encoding for the above-described cassettes were added. At 72 hours post infection, the media was replaced by fresh media (100 μl per well) containing 1 mg/ml MTT and the cells were incubated for 2–4 more hours. MTT-formazan crystals were dissolved by the addition of extraction solution (0.1N HCl in absolute isopropanol). Absorbance at 570 nm and a reference wavelength of 690 nm were recorded on an automated microplate reader.

### Detection of cell death

#### Apoptosis

Cells were seeded in 12-well plates (1 × 10^5^ cells/well) in complete medium and infected with the different adenoviruses at several multiples of infection (MOI) for 72 hours. Annexin V (Annexin V, CF640R conjugate) was detected according to the manufacturer's protocol (Biotium Inc., USA). The cells were washed with PBS and then incubated in a solution of Annexin V binding protein. The cells were subsequently analyzed by flow cytometry [FACSCalibur (Becton Dickinson, CA)], and the results were examined with the CELLQuest program (Becton Dickinson).

### Total dead cells

Cells were seeded in 12-well plates (1 × 10^5^ cells/well) in complete medium and infected with the different adenoviruses at several MOI for 72 hours. Dead cells were detected by RedDotTM2, a far-red cell membrane-impermeable nuclear dye, according to the manufacturer's protocol (Biotium Inc., USA). The cells were washed with PBS, and then incubated in a solution of RedDot2 dye. Far red nuclear staining was detected by flow cytometry.

### End-point dilution assay (EPDA)

1 × 10^4^ HEK293 cells/well were seeded in 96-well plate in 100 μl of growth medium. The recombinant adenovirus stock solutions were serially diluted 10-fold to a concentration in a range of 10^−3^−10^−10^ into growth medium and added to each well in columns 1–10. Virus-free growth medium was added to the wells in columns 11 and 12 which served as controls for the viability of non-infected cells. The plate was incubated in a humidified CO_2_ (5%) incubator for 10 days at 37°C. Each well was checked for CPE using a microscope. A well was scored as CPE positive even if only a few cells showed cytopathic effects. The viral titer was calculated according to the formula: Titer (pfu/ml) = 10^(x + 0.8)^, where x = the sum of the fractions of CPE-positive wells for each dilution (10 out of 10 wells with CPE calculated as “1”).

### Colony formation assay

5 × 10^5^ HCT116 and HT29 cells were seeded per well in 6-well plates. After 24 hours, the cells were infected with 25 and 10 MOI of the viruses “pAdEasy-Py4-mCherry-MazF-IRES-TetR-CMVmp-MazE-IRES-EGFP“ and “pAdEasy-SV40-mCherry-MazF-IRES-TetR-CMVmp-MazE-IRES-EGFP”, or left uninfected. After 7 hours, the cells were trypsinized and seeded in 3-fold dilutions and incubated for 7 days. Surviving colonies were fixed with 4% formaldehyde in PBS and stained with 0.02% crystal violet.

### Xenograft model in mice for measuring *in vivo* tumor development

Male 6–8 week old athymic nude mice (Harlan Laboratories) (*n* = 18) were housed in sterile cages and handled with aseptic precautions. The mice were fed ad libitum. For testing the therapeutic potential of the TA system, exponentially growing HCT116 cells were harvested and resuspended at a final concentration of 5 × 10^6^ cells per 0.1 ml PBS per injection. The cells were injected subcutaneously at two sites on the backs of the mice. When tumors were palpable (~0.3–0.5 cm^3^), the mice were randomly divided into three groups of six and the treatment was initiated. The viruses Ad-Py4-TA (6 mice) and ΔPy4-TA 1 × 10^9^ pfu (6 mice) or PBS (6 mice) were administrated via two intraperitoneal injections with a 3-day interval between injections. The mice were weighed, the tumor volume was measured with a caliper every two days starting from treatment onset, and the results were carefully plotted. Tumor volume was calculated as 4/3π∙a∙b^2^. At the end of the experiment, MazF and MazE expression in the tumors was monitored by imaging using the CRi Maestro system. The mice were anesthetized and then sacrificed by cervical dislocation and the tumors were excised. The study was approved by the Institutional Review Board of Tel Aviv Sourasky Medical Center.

### Statistics

Data from the *in vitro* studies are presented as a mean ± SD of sets of data as determined in triplicates. Statistical significance between treatments was determined by Student *t-test*, *P* values < 0.05 were considered significant.

In the *in vivo* studies, the tumor-bearing mice were randomized into various treatment groups (*n* = 6) and the tumor volumes were periodically monitored and calculated as 4/3π∙a∙b^2^. Statistical significant differences between groups and at different time points were determined by Student *t-test*.

### Study approval

The study was approved by the Institutional committee for animal welfare at Tel-Aviv Sourasky Medical Center.

## SUPPLEMENTARY MATERIALS


