# Supplementary material for: Selective eradication of cancer cells by delivery of adenovirus-based toxins
Source: Oncotarget. 2017 Apr 7;8(24):38581–91. doi: 10.18632/oncotarget.16934 (PMC5503555; doi:10.18632/oncotarget.16934)
Supplement: Supplementary file 1 [file oncotarget-08-38581-s001.pdf]

Selective eradication of cancer cells by delivery of adenovirus-based toxins

Supplementary Materials

Supplementary Text 1

Construction of the vector encoding for "mCherry-MazF"

The monomeric red fluorescence protein mCherry was amplified from an expression cassette by PCR. The PCR product was digested with *Hin*dIII and *Xba*I and cloned between the corresponding sites of the plasmid "pGL3 promoter-Py4-PUMA" (replacing the *PUMA* gene) that had been previously prepared, generating the "pGLl3 promoter-Py4-mCherry" plasmid.

The MazF coding sequence which was amplified from an expression cassette (kindly provided by Dr. Assaf Shapira, Department of Molecular Microbiology and Biotechnology, Tel-Aviv University, Israel) using the primes Hind-Cher-For and Xba-Maz-Rev ("5′- CT TTTGCAAAAAGCTTCCACCATGggaattcacGTGAGCA AGGGCGAGGAGG-3′ and 5′- CCGCCCCGACTCTA GActaaccggtgccaatcagtacgttaattttggc-3′, respectively) and was fused to the C terminus of the mCherry under the RRE, Py4-SV40 mP, generating the "pGL3 promoter-Py4-mCherry-MazF" plasmid. This intermediate vector was used as the template for the amplification of the Py4-mCherry-MazF fusion genes, and the amplified product was cloned using the AdEasy system (pShuttle and pAdEasy-1), as previously described [1, 2], to generate the regulated expression cassette "pAdEasy-Py4-mCherry-MazF".

Construction of the vector encoding for "mCherry"

The sequence of the red fluorescent protein mCherry [3] was amplified by PCR from an expression cassette and cloned downstream to a CMV promoter, generating the expression cassette "pAdEasy-CMV-mCherry".

Construction of the vector encoding for "MazEF"

The tetracycline repressor coding sequence was located downstream to an IRES sequence and cloned downstream to the SV40-mCherry-MazF sequence. An additional arm was introduced to this cassette, controlled by a different minimal promoter (mP) without the RAS-responsive elements. Starting from its N terminus, this arm includes: a CMV mP, a tetracycline operator sequence, the full MazE coding sequence, IRES and EGFP coding sequence (Figure 2A), generating the expression cassette "pAdEasy-Py4-mCherry-MazF-IRES-TetR-CMVmp-MazE-IRES-EGFP"

Plasmids were isolated by a standard miniprep procedure and sequenced to confirm their predicted composition. For the production of virus particles, the plasmids "pAdEasy-Py4-mCherry-MazF", "pAdEasy-Py4-mCherry-MazF-IRES-TetR-CMVmp-MazE-IRES-EGFP","pAdEasy-SV40-mCherry-MazF-IRES-TetR-CMVmp-MazE-IRES-EGFP " and "pAdEasy-mCherry" were isolated from selected "positive" clones and digested with *Pac*I. Next, DNA was purified using a Zymoclean™ Gel DNA Recovery Kit according to the manufacturer's instructions. Five µg of the purified, digested plasmids were used to transfect 70% confluence HEK293 cells (supplemented with tetracycline at a final concentration of 1 µg/ml when the TA construct had been transfected) and HEK293-MazE cells (for the mCherry-MazF construct, supplement S2) in 60-mm culture plates using the calcium-phosphate method. After 24 hours, the transfection medium was replaced with 5 ml of fresh medium (that was supplemented with 1 µg/ml of tetracycline in the TA system). From 7 to 10 days post-transfection, when a cytopathic effect (CPE) had been clearly observed, the cells were collected by scraping them off the plate and pelleting them along with any floating cells in the culture. The pellet was washed once with phosphate-buffered saline (PBS), suspended in 0.5 ml PBS and subjected to 4 cycles of freeze/thaw. Cell debris was precipitated by brief centrifugation, and 300 µl of the supernatant that contained virus particles were used to infect 70% confluent HEK293 and its derivative cells in 10 cm plates (first amplification "cycle"). When one-third to one-half of the cells had been detached (usually after 3–5 days), virus particles were released by freeze/thaw cycles as described above. The supernatant containing viruses was kept at –80ºC.

High scale production of the adenoviruses was performed by SIRION biotech, Germany.

Supplementary Text 2

Establishment of new packaging cell line for the production of the toxin

The lethal transgene MazF coding sequence was fused to the mCherry gene and cloned into the Ad5 adenoviral vector plasmid DNA. The propagation of the

virus particles in the HEK293 packaging cells resulted in low yields of virus production due to the highly toxic nature of the selected gene. Hence, an innovative packaging system was established, based on MazF-resistant HEK293 cells that constitutively express the MazE antitoxin, encoded from the pIRES2-MazE-IRES-EGFP plasmid. The EGFP marker was used for effective and rapid screening of stable clones according to their high fluorescence intensity. The yield was higher by almost 2 orders of magnitude after propagation in MazF-resistant cells compared to propagation in parental HEK293 packaging cells.

REFERENCES

1. He TC, Zhou S, da Costa LT, Yu J, Kinzler KW, Vogelstein B. A simplified system for generating recombinant adenoviruses. Proc Natl Acad Sci USA. 1998; 95:2509–14.
2. Luo J, Deng ZL, Luo X, Tang N, Song WX, Chen J, Sharff KA, Luu HH, Haydon RC, Kinzler KW, Vogelstein B, He TC. A protocol for rapid generation of recombinant adenoviruses using the AdEasy system. Nat Protoc. 2007; 2:1236–47.
3. Shaner NC, Campbell RE, Steinbach PA, Giepmans BN, Palmer AE, Tsien RY. Improved monomeric red, orange and yellow fluorescent proteins derived from Discosoma sp. red fluorescent protein. Nat Biotechnol. 2004; 22:1567–72.
